# Gelatin Sponge-Embedded Adipose-Derived Stromal Cells Enable Allogeneic Application for Revascularization of Ischemic Wounds

**DOI:** 10.3390/ijms27083482

**Published:** 2026-04-13

**Authors:** Manon Locatelli, Wolf-Henning Boehncke, Damien Pastor, Jean Villard, Nicolo-Constantino Brembilla, Olivier Preynat-Seauve

**Affiliations:** 1 Department of Medicine, Faculty of Medicine, University of Geneva, 1206 Geneva, Switzerland; manon.locatelli@unige.ch (M.L.); jean.villard@hug.ch (J.V.); 2 Division of Dermatology and Venerology, Geneva University Hospitals, 1205 Geneva, Switzerland; wolf-henning.boehncke@hug.ch (W.-H.B.); damien.pastor@hug.ch (D.P.); nicolo.brembilla@unige.ch (N.-C.B.); 3 Platform of Clinical Cell Therapy, Department of Diagnostics, Geneva University Hospitals, 1205 Geneva, Switzerland; 4 Heketiss SA, Plan-Les-Ouates, 1228 Geneva, Switzerland

**Keywords:** adipose-derived stromal cells, wound healing, chronic wounds, allogeneic cell therapy, angiogenesis

## Abstract

Chronic wounds are ulcers unable to heal due to vascular insufficiency, diabetes, or obesity. Adipose-derived stromal cells (ASCs) are promising candidates for regenerative therapies owing to their pro-healing and angiogenic properties. Compared with autologous approaches, allogeneic ASC therapies offer the opportunity for off-the-shelf use, enabling immediate availability, standardized qualification, and consistent potency. Gelatin sponges have been shown to reprogram ASCs toward a highly angiogenic phenotype. However, because this activation also modulates some immune-related genes, including MHC, its impact on immunogenicity is unknown and could be critical for allogeneic applications. This study evaluated whether ASCs embedded in a gelatin sponge could be used in an allogeneic setting for ischemic wound repair. To mimic clinical allogeneic conditions, a controlled MHC mismatch was introduced in a rat ischemic wound model: donor ASCs carrying RT1^n or RT1^l haplotypes were implanted into outbred RT1^a recipients. Embedding ASCs within the gelatin sponge upregulated MHC class I but not class II expression, without inducing systemic or local alloreactivity. Serum acute-phase proteins remained unchanged, and no CD3^+^ T-cell infiltration was detected. Histology confirmed efficacy on ischemic wounds, with increased granulation tissue thickness, red blood cell infiltration, and enhanced vessel density versus controls. Allogeneic ASCs activated by a gelatin scaffold promote wound revascularization without eliciting immune rejection, supporting their development as standardized, off-the-shelf therapies for chronic ischemic wounds.

## 1. Introduction

Chronic ischemic wounds, such as diabetic foot ulcers and venous leg ulcers [1], represent a major and growing public health burden [2,3,4]. Chronic wounds arise from distinct yet overlapping pathophysiological mechanisms. In diabetes, arterial disease and microvascular dysfunction reduce tissue perfusion and oxygen delivery, leading to ischemia. Obesity further compromises wound healing by promoting chronic low-grade inflammation, altered adipokine signaling, and impaired cellular responses required for tissue repair. In venous insufficiency, sustained venous hypertension and compression impair capillary exchange, resulting in edema, tissue hypoxia, and inflammatory cell trapping. Thus, these wounds are characterized by insufficient vascularization, persistent hypoxia, chronic inflammation, and impaired healing—frequently culminating in infection, limb amputation, and elevated healthcare costs [5]. Despite advances in wound care, a significant subset of patients remains unresponsive to current treatments. In these refractory cases, the central pathophysiological barrier is the inability to restore vascular perfusion in the wound bed—a critical prerequisite for effective healing [2,5].

Adipose-derived stromal cells (ASCs), a subtype of mesenchymal cells in adipose tissues [6], have emerged as promising candidates for regenerative therapies targeting chronic wounds [2]. ASCs are abundant, easily harvested via minimally invasive procedures, and capable of secreting a wide secretome of bioactive factors with pro-angiogenic, immunomodulatory, and extracellular matrix remodeling functions [7,8,9,10,11]. Among these, their secretion of angiogenic mediators plays a pivotal role in promoting endothelial activation, neo-vessel formation, and tissue revascularization [12,13,14]. This paracrine-driven mechanism is particularly suited to overcoming the vascular insufficiency that defines refractory ischemic wounds.

Our research previously demonstrated that ASC can be functionally switched towards a potent angiogenic state through integrin engagement by RGD (Arg-Gly-Asp) motifs [15]. It has been shown that proper RGD exposure markedly enhances ASC secretion of key vascular mediators and stimulates endothelial cell migration, tubulogenesis, and vascular stability in vitro. Increased vasculogenic potential was also observed in pluripotent stem cell-derived vascular organoids [15]. To translate this mechanism into a therapeutic option, a strategy has been developed to properly deliver RGD signals to ASC using a clinically approved, crosslinked porcine gelatin sponge. Under optimized experimental culture conditions, this scaffold effectively delivers RGD motifs to ASC, maintaining over time their phenotype and viability, triggering robust angiogenic secretions [15,16]. In an in vivo preclinical study [16], rat ASCs were embedded within an RGD-exposing crosslinked gelatin scaffold and applied to a rat model of chronic ischemic footpad wounds. This approach demonstrated that ASC in the gelatin sponge significantly improved wound revascularization and healing compared to controls.

However, the study was conducted in outbred Wistar rats sharing the same MHC haplotype (RT1^a), thereby more closely modeling a non-consanguine setting that does not modelize the immunological challenges of a real allogeneic cell therapy in humans.

The development of allogeneic ASC-based therapies is a critical step toward clinical translation. Indeed, autologous ASC approaches are strongly limited by donor-to-donor variability, the heterogeneity between harvesting techniques [17] and fat sampling [18,19,20], delayed production timelines, high costs, and the well-documented impairment of ASC function in elderly, diabetic, or ischemic patients [21,22,23]. In contrast, allogeneic approaches based on standardized and pre-qualified ASC banks enable scalable manufacturing and consistent therapeutic performance [2].

Supporting this strategy, ASCs are known to exhibit low immunogenicity and immunosuppressive properties [24,25,26,27], making them suitable for allogeneic applications. Several clinical trials have already tested allogeneic ASC—though not combined with a pro-angiogenic biomaterial scaffold—in the treatment of chronic wounds, demonstrating favorable safety profiles and preliminary efficacy [16]. These studies support the feasibility of using ASC in an allogeneic setting for regenerative medicine. However, while the baseline immunological properties of allogeneic ASCs are relatively well characterized, the impact of specific activation strategies—such as RGD-mediated angiogenic activation—on their efficacy on chronic wounds remains unknown. Given that RGD exposure, through gelatin sponges, profoundly modulates ASC functions [15,16], including increased MHC I expression and pro-inflammatory secretions, which could possibly induce alloreactivity. Thus, it is essential to determine whether this RGD-activated state alters their immunogenicity in an allogeneic context.

The rat MHC complex, namely RT1, displays major polymorphism across distinct haplotypes characteristic of different strains, including RT1^a (Wistar), RT1^l (Lewis), and RT1^n (Brown Norway). These haplotypes vary in the number, composition, and sequence of both classical class I (RT1-A, RT1-C/E) and class II (RT1-B, RT1-D) loci, each carrying unique allelic variants and gene copy numbers within these clusters [28,29,30]. Consequently, combinations of donors and recipients carrying different RT1 haplotypes generate MHC mismatches, leading to predictable allogeneic incompatibility and thereby providing a relevant model for allogeneic cell therapy.

In the present study, we assessed the efficacy and immune safety of allogeneic ASC embedded in an RGD-exposing gelatin scaffold using a rat model of ischemic footpad wounds incorporating a controlled MHC haplotype mismatch between donors and recipient animals.

## 2. Results

### 2.1. Rat ASCs in a Gelatin Sponge Up-Regulate the Expression of MHC I

The use of a gelatin sponge has previously been shown to effectively expose RGD motifs to human or rat ASCs, leading to profound gene expression changes and the induction of a highly angiogenic state [15]. Since this RGD-mediated regulation markedly alters the gene expression profile, including the modulation of MHC molecules and inflammatory factors, the immunogenicity of ASCs embedded in a gelatin sponge may differ from that of ASCs in conventional culture, particularly in an allogeneic context.

**Figure 1:**
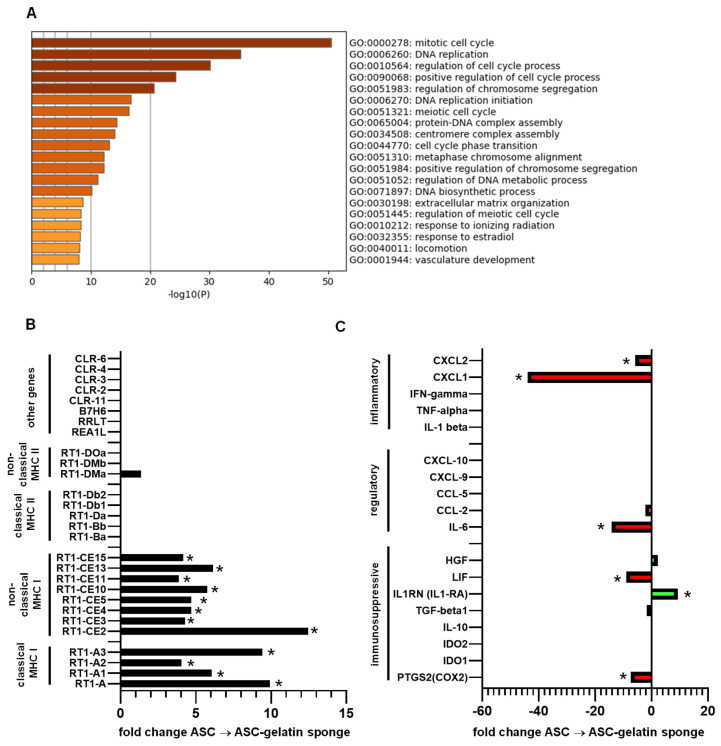
Rat ASCs (RT1^a) within a gelatin sponge modify their transcriptome and MHC molecules. The transcriptome of rat ASC in gelatin generated from 3 independent cell lines was assessed by microarray and compared to rat ASC from the same 3 donors grown in a monolayer. (**A**)—Enrichment pathways determined using Metascape and the Gene Ontology Biological Process (GOBP) gene set from the Molecular Signature. (**B**)—Fold change analysis on MHC I and MHC II genes/paralog genes of the rat RT1 system. Genes with an empty histogram were not detected. (**C**)—Fold change analysis on immune-related genes. Genes with an empty histogram were not detected. * *p* < 0.05 (moderated *t* test, Benjamini–Hochberg FDR adjustment).

The first set of experiments was conducted in rats, using rat ASC derived from healthy adipose tissue and cultured in a conventional medium supplemented with human platelet lysate and fetal bovine serum. Culturing rat ASCs within a gelatin sponge, similarly to human ASCs, strongly modified their global gene expression profile, shifting it toward a regenerative and pro-angiogenic phenotype [16]. Functional annotation of the most significantly regulated genes confirmed a marked impact on ASC proliferation and angiogenic capacities (Figure 1A). Given that polymorphic MHC molecules are the main contributors to alloreactivity against allogeneic T cells, we first investigated whether embedding rat ASCs within a gelatin sponge scaffold modulates their expression, as well as other ligands linked to alloreactivity. To this end, ASCs purified from rat adipose tissue were cultured under two conditions: (i) standard two-dimensional monolayer culture and (ii) within a gelatin sponge scaffold, corresponding to the patch used for the treatment of ischemic wounds. Differential gene expression analysis revealed that rat ASCs embedded in the gelatin sponge displayed a significant up-regulation of several transcripts encoding MHC class I and class II molecules. Multiple paralog genes of the highly polymorphic RT1-A classical MHC class I cluster were up-regulated, together with RT1-C/E genes belonging to non-classical MHC class I families (Figure 1B). In contrast, classical polymorphic MHC class II genes (RT1-B and RT1-D) as well as non-classical MHC class II genes (RT1-DM and RT1-DO) were neither expressed nor regulated by gelatin sponge culture. Other rat genes involved in alloreactivity were not expressed. In addition to ligands involved in the allorecognition, genes linked to immunomodulation and inflammation were also compared. If some regulations were noticed, notably the reduced pro-inflammatory CXCL-1, no signs of regulations in favor of alloreactivity were observed (Figure 1C). Together, these observations indicate that culturing ASCs within a gelatin sponge induces the upregulation of some MHC class I genes involved in allorecognition, as well as some less polymorphic class I genes, without significant regulation of inflammatory secretions, supporting that the immunogenicity of ASCs could be modified in vivo in an allogeneic setting.

### 2.2. Experimental Rat Model for Mimicking Allogeneic ASC - Gelatin Sponge Therapy in Ischemic Wounds

**Figure 2:**
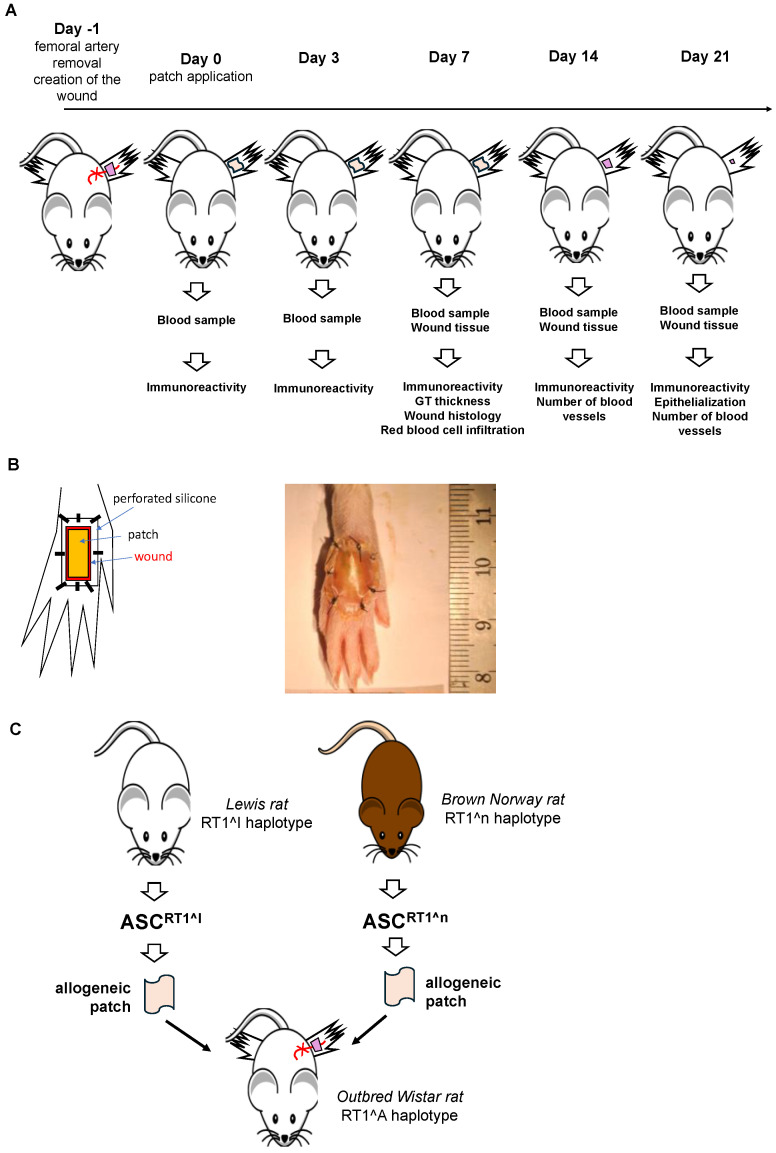
Experimental setup to evaluate the preclinical efficacy of allogeneic ASC/gelatin sponge treatment in rats. (**A**)—Allogeneic ASC/gelatin sponges, empty gelatin sponges, or standard silicone/polyurethane dressings were sutured onto full-thickness wounds created on the ischemic hind foot of Wistar rats. Treatments were maintained in place for 21 days, with various experimental readouts collected at defined time points. (**B**)—Schematic representation and photograph illustrating the stabilization of the patches on the wound. (**C**)—Experimental design to establish allogeneicity between donor ASCs and recipient animals. A controlled MHC mismatch was achieved by using donors and recipients with different MHC haplotypes. To further include minor antigen polymorphisms, outbred rats were used as recipients. The outbred Wistar colony used here exhibited additional minor antigen polymorphism within the RT1^a background, further increasing genetic diversity among recipients.

A preclinical model was established to replicate the conditions of allogeneic ASC therapy for ischemic wound treatment, using a combination of surgically induced hindlimb ischemia and a standardized cutaneous wound. Female Wistar rats were selected as recipients due to their expression of the RT1^a haplotype, mixing defined MHC I and MHC II alleles. They were non-consanguine outbred rats, thus adding minor polymorphisms between individuals to increase immunogenetic heterogeneity. To induce sustained ischemia, a 1 cm segment of the femoral artery was excised, ensuring disruption of arterial inflow to the distal limb. Ischemia was consistently achieved using the established rat foot wound model, as previously described [31]. Successful ischemia was confirmed by the absence of bleeding at the time of wound creation, a marked delay in wound closure compared with non-ischemic controls, and a characteristic pale skin appearance indicative of reduced tissue perfusion. Immediately after arterial resection, a standardized full-thickness excisional wound was created on the dorsal surface of the ipsilateral foot, removing the skin down to—but not including—the underlying fascia. This dual injury model (arterial excision plus skin defect) produced a reproducible ischemic wound environment that substantially delayed spontaneous wound closure [31]. Each recipient rat received a gelatin sponge—an ASC patch one day later, positioned to cover the entire wound surface. A scheme of the experimental setup is shown in Figure 2A. The patches were stabilized on the wound using a gas-permeable silicone gutter, which was sutured onto the wound (Figure 2B). To maintain a moist environment and support optimal wound healing, a gas-permeable polyurethane dressing was added. A 24 h interval was introduced between skin excision and treatment application to ensure that the acute defect transitioned into a wound. This delay allowed the initiation of early healing processes, particularly inflammation, which are essential features of an ischemic wound. The silicone gutter and sutures were removed on day 7 to allow for the natural progression of wound healing (Figure 2A). The systemic immune reactivity markers were measured at days 3, 7, 14, and 21 through blood collection. To ensure clinically meaningful evaluation of wound revascularization, the efficacy endpoints were performed through wound fixation and histological assessment: granulation tissue formation during the early healing phase (day 7), early red blood cell infiltration in granulation tissue before neo-vessel formation as an indicator of early tissue oxygenation (day 7), and vascular density in later stages of healing (day 14 and day 21) (Figure 2A). Granulation tissue formation, early tissue oxygenation, and vascularization are indeed crucial for wound healing. This is especially relevant in ischemic wounds, such as diabetic foot ulcers and venous ulcers, where impaired blood supply and delayed tissue regeneration are the cause of the disease. To replicate the allogeneic context in this preclinical setting, the rat model of ischemic wound was also designed to incorporate controlled MHC mismatches between the donor ASCs and the recipients. Recipient animals were outbred Wistar rats carrying the RT1^a haplotype, while donor ASCs were obtained from Lewis (RT1^l haplotype) or Brown Norway (RT1^n haplotype) rats (Figure 2C). As each haplotype reflected a distinct combination of alleles across the entire RT1 complex, donors and recipients then differed in both polymorphic MHC class I and MHC class II regions [32,33,34]. Thus, this design generated defined, reproducible MHC I/II mismatches between donor and recipient strains, comparable to the unavoidable HLA disparities observed in human allogeneic cell therapy when a banked product from a single donor is administered to unrelated recipients.

### 2.3. Rats Treated by Allogeneic ASC–Gelatin Sponge Patches Did Not Show Systemic and Local Signs of Alloreactivity

**Figure 3:**
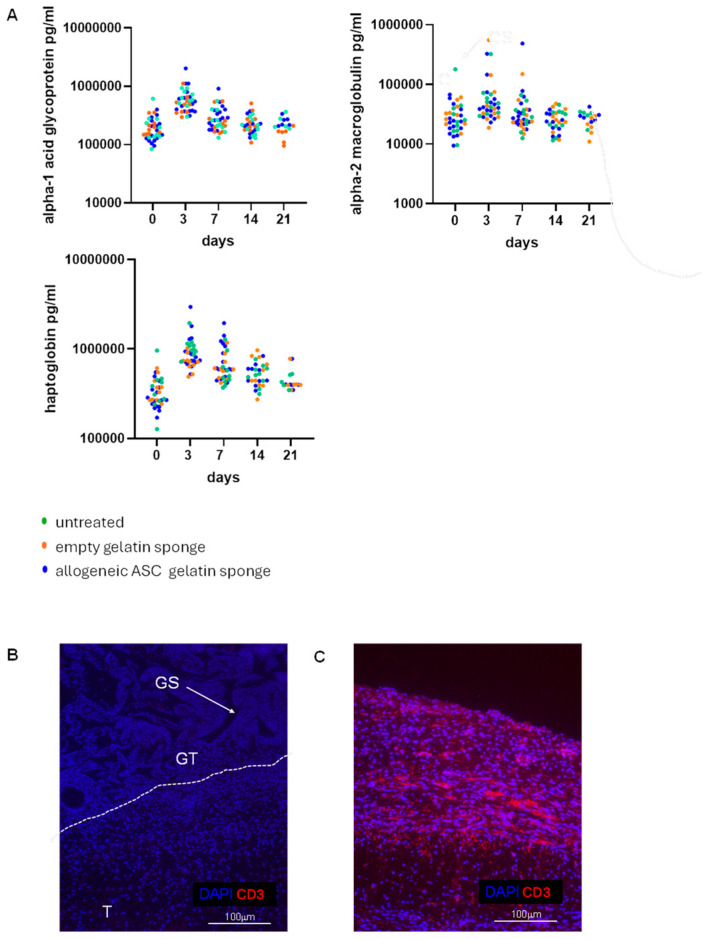
Assessment of systemic markers of immune reactivity in rats treated with allogeneic ASC-gelatin sponge patches. Allogeneic ASC/gelatin sponges (RT1^l⟶RT1^a *n* = 7; RT1^n⟶RT1^a *n* = 7), empty gelatin sponges (*n* = 10), or standard silicone/polyurethane dressings (untreated, *n* = 10) were sutured onto full-thickness wounds created on the ischemic hind foot of Wistar rats. Treatments were maintained in place for 21 days, with serum collection at defined time points. (**A**)—Systemic markers of immune reactivity were measured in sera by Luminex Discovery Assay. (**B**)—7 immunofluorescent staining (CD3) of a histological section of the healing area at day 7. GT: Granulation Tissue, GS: Gelatin Sponge, T: flexor digitorum Tendons. (**C**)—7 immunofluorescent staining (CD3) of a histological section of an area of inflammatory rat skin, used as a technical positive control for antibody staining. DAPI = 4’,6’-diamidino-2-phenylindole.

As part of the immunological safety assessment, three systemic inflammatory markers associated with immune reactivity and alloreactivity were quantified in serum from treated rats and compared them with those from animals receiving empty gelatin sponges or no treatment. The selected markers—alpha-1-acid glycoprotein, alpha-2-macroglobulin, and haptoglobin—are established acute-phase proteins whose elevations have been documented in rodent transplantation models as indicators of alloreactivity [35,36,37,38]. Measurements were performed at different post-treatment time points, from day 0 to day 21, to detect potential immune activation triggered by the implanted ASC and/or gelatin sponge. No significant differences in marker levels were observed between the untreated group, the allogeneic ASC–gelatin sponge group, and the empty gelatin sponge group at either time point (Figure 3A). A transient increase in all three markers was noted at day 3 across all experimental groups, including untreated controls, suggesting that this response was related to the surgical procedure and wound creation rather than to the presence of allogeneic ASCs. In parallel, immunohistochemistry analysis revealed no CD3^+^ T-cell infiltration in the gelatin sponge of animals treated with allogeneic ASCs in gelatin sponge (Figure 3B). Collectively, these findings indicate that the implantation of allogeneic ASCs within a gelatin sponge does not elicit detectable systemic immune reactivity or immune rejection in this model, supporting its progression to efficacy evaluation.

### 2.4. At Day 7, the Allogeneic ASC–Gelatin Sponge Acts as a Scaffold Infiltrating a Thicker and Red Blood Cell-Enriched Granulation Tissue

To ensure a controlled experimental setting, the following treatment conditions were compared: (i) allogeneic ASC embedded in a gelatin sponge scaffold, (ii) empty gelatin sponges (without ASC), given that gelatin itself provides scaffold properties and has intrinsic biological effects on wound healing, to distinguish the specific contribution between ASC and the scaffold, (iii) untreated control group to further evaluate the natural wound healing process, where the wound was covered only with a polyurethane film, without any sponge or ASC patch.

**Figure 4:**
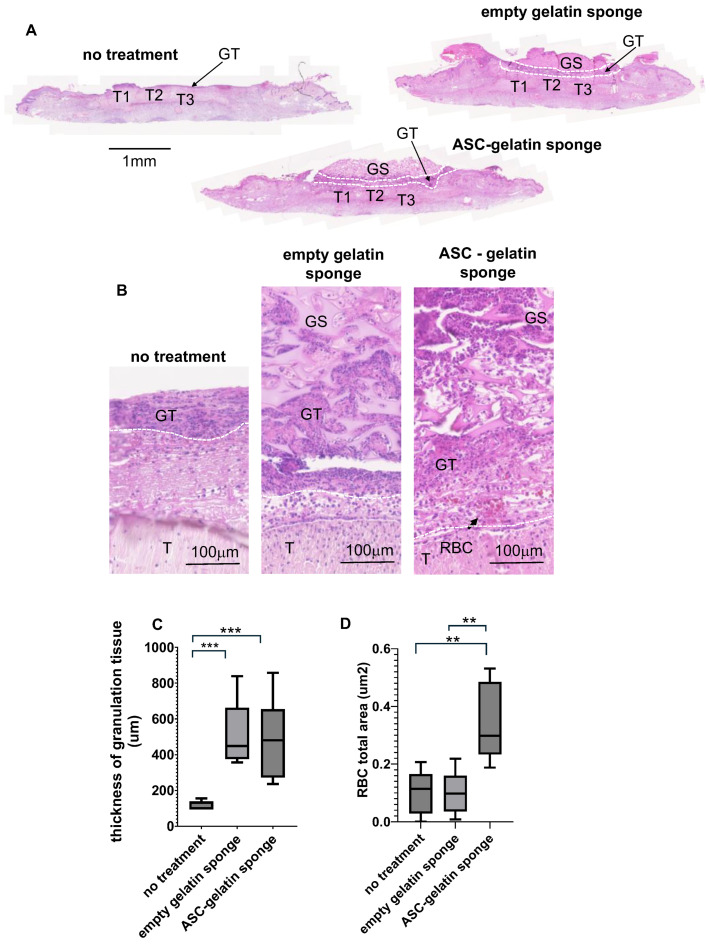
Histological section of the wound area at day 7 post-treatment. Allogeneic ASC/gelatin sponges (RT1^l⟶RT1^a *n* = 3; RT1^n⟶RT1^a *n* = 3), empty gelatin sponges (*n* = 7), or standard silicone/polyurethane dressings (untreated, *n* = 7) were sutured onto full-thickness wounds created on the ischemic hind foot of Wistar rats. (**A**)—Hemalun/eosin coloration was performed in histological sections of the healing area at day 7. T indicates flexor digitorum tendons. GT indicates Granulation Tissue. GS indicates Gelatin Sponge. (**B**)—Hemalun/eosin coloration was performed in histological sections of the healing area at day 7 (higher magnification). (**C**)—Granulation tissue thickness is measured by the software-assisted picture analysis Zen 3.11 (Zeiss). The box represents the interquartile range (IQR), spanning from the first quartile (Q1, 25th percentile) to the third quartile (Q3, 75th percentile). The horizontal line inside the box indicates the median (Q2, 50th percentile). Whiskers extend to the smallest and largest values. (**D**)—The red blood cell area was determined by software-assisted analysis (QPath 0.5.1).** *p* < 0.01; *** *p* < 0.005 (Mann–Whitney, GraphPad Prism 10).

At day 7 post-treatment, wound healing in rodents is characterized by the formation of disorganized granulation tissue, primarily composed of red blood cells (RBCs), leukocytes, and fibroblastic cells. At this stage, very few or no organized tubular vascular structures are present within the newly formed tissue. The wound was created at the center of the rat footpad by excising the skin covering three flexor digitorum tendons. Consequently, histological evaluations were performed in the healing zone, located immediately beyond and delineated by these tendons. Within the healing area, several cell populations characteristic of rat granulation tissue were observed based on their morphology: red blood cells, fibrocytic cells (eosin-stained with fibroblastic morphology), neutrophils, and monocytic cells/macrophages (according to their typical H&E morphological features). In the untreated control group, a thin granulation tissue (GT) layer was observed, as evidenced by a dense hemalun-positive (violet-stained) area beyond the tendons (T) (Figure 4A). In contrast, in both the empty gelatin sponge (GS) and allogeneic patch groups, the gelatin sponge was densely infiltrated by thicker granulation tissue, indicating a more advanced wound healing response (Figure 4A). Higher magnification histological sections confirmed these findings: in both the empty gelatin sponge and ASC patch conditions, the granulation tissue exhibited dense infiltration of leukocytes, eosinophilic fibroblastic cells, and RBCs, which were distributed between the eosin-colored gelatin trabeculae (Figure 4B). Software-assisted quantitative analysis confirmed that the ASC-gelatin sponge and the empty gelatin sponge acted as scaffold structures, integrating within the healing zone and promoting the development of a thicker granulation tissue compared to untreated animals (Figure 4C). At this point, the presence of ASC within the gelatin sponge did not influence granulation tissue thickness. Also, at this early stage of healing, vascularization remains immature, with no/limited evidence of organized blood vessel formation in granulation tissue. However, RBC infiltration, an essential early indicator of tissue oxygenation, was observed. ASC-gelatin sponge-treated wounds exhibited a higher degree of RBC infiltration compared to both the empty gelatin sponge and untreated control groups (Figure 4B). Software-assisted quantification confirmed that, under ischemic conditions, the patch significantly increased RBC infiltration within the granulation tissue seven days post-application (Figure 4D). This effect was attributed to the presence of ASC, which appeared to facilitate RBC colonization rather than being a result of bleeding, as the empty gelatin sponge did not induce a similar response.

### 2.5. At Day 14, the Allogeneic ASC–Gelatin Sponge Did Not Increase the Density of Blood Vessels Compared to the Empty Gelatin Sponge and Untreated Animals

**Figure 5:**
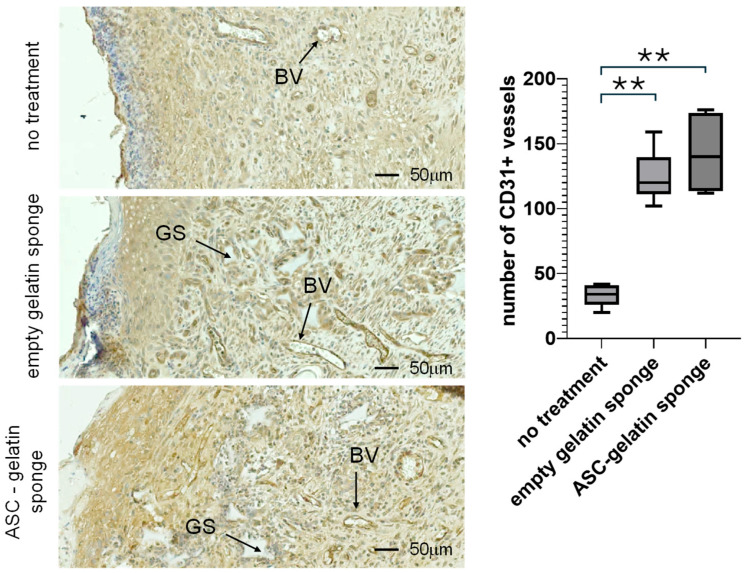
Histological section of the wound area at day 14 post-treatment. Allogeneic ASC–gelatin sponges (RT1^l⟶RT1^a *n* = 3; RT1^n⟶RT1^a *n* = 2), empty gelatin sponges (*n* = 4), or standard silicone/polyurethane dressings (untreated, *n* = 5) were sutured onto full-thickness wounds created on the ischemic hind foot of Wistar rats. CD31 immunostaining was performed in histological sections of the healing area at day 14 post-treatment. GS indicates Gelatin Sponge, and BV indicates Blood Vessels. The number of blood vessels was determined by software-assisted analysis (Zeiss Zen 3.11 software). The box represents the interquartile range (IQR), spanning from the first quartile (Q1, 25th percentile) to the third quartile (Q3, 75th percentile). The horizontal line inside the box indicates the median (Q2, 50th percentile). ** *p* inf 0.01 (Mann–Whitney).

By day 14, wound healing in rodents is characterized by the maturation of a more organized granulation tissue, with a progressive decline in inflammation and leukocyte infiltration, and a marked increase in fibroblast proliferation. The granulation tissue observed at day 14 became progressively less compact, consistent with the transition toward the tissue remodeling phase. At this later stage of healing, blood vessels (BV) are formed near the wound area; therefore, immunostaining for CD31 was performed to assess neovascularization. The gelatin sponge scaffold was observed to be partially resorbed, with an observed reduced density and size of gelatin trabeculae in the empty sponge and ASC–gelatin sponge groups, likely due to the action of wound-associated collagenases (Figure 5). Although residual granulation tissue at day 14 remained partially disorganized and inflammatory, particularly near the air-exposed surface, tubular CD31-positive endothelial structures were detectable, enabling the quantification of newly formed BVs. CD31^+^ BVs were present in all groups, with a significant increase in vessel density in both gelatin sponge–treated groups compared with the untreated group (Figure 5). However, no statistically significant difference was observed between the empty sponge and ASC–gelatin sponge groups, suggesting that, at this stage, the pro-angiogenic effect was attributable primarily to the gelatin scaffold rather than to the presence of ASCs.

### 2.6. At Day 21, the Allogeneic ASC–Gelatin Sponge Increases the Density of Blood Vessels Compared to Empty Gelatin Sponge and Untreated Animals

**Figure 6:**
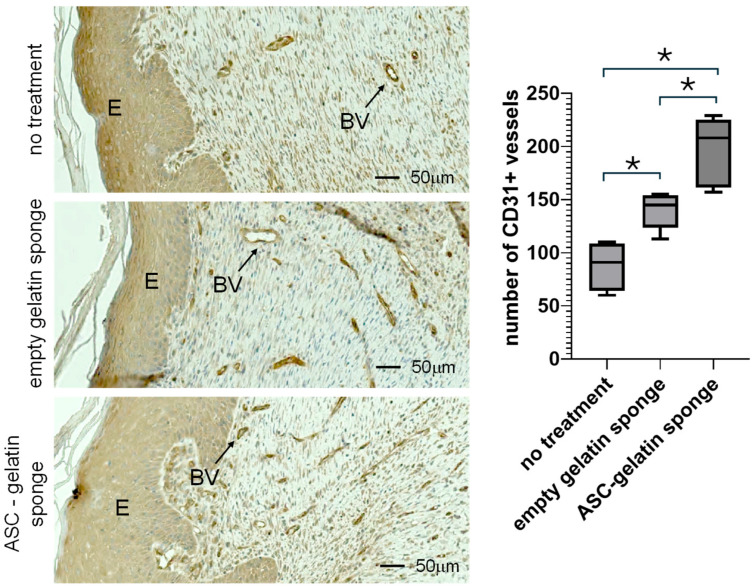
Histological section of the wound area at day 21 post-treatment. Allogeneic ASC–gelatin sponges (RT1^l⟶RT1^a *n* = 3; RT1^n⟶RT1^a *n* = 4), empty gelatin sponges (*n* = 6), or standard silicone/polyurethane dressings (untreated, *n* = 5) were sutured onto full-thickness wounds created on the ischemic hind foot of Wistar rats. CD31 immunostaining was performed. E indicates the newly formed epidermis. BV indicates blood vessels. The number of blood vessels was determined by software-assisted analysis (Zeiss Zen 3.11 software). The box represents the interquartile range (IQR), spanning from the first quartile (Q1, 25th percentile) to the third quartile (Q3, 75th percentile). * *p* inf 0.05 (Mann–Whitney).

By day 21, wound healing in rodents is characterized by the formation of a more structured and organized tissue, with a strong reduction in leukocyte infiltration and a significant increase in epithelial maturation. The newly formed tissue in the allogeneic ASC–gelatin sponge-treated group appeared structurally well-organized, comparable to the empty gelatin sponge and untreated control groups. The gelatin sponge scaffold was observed to be fully resorbed, with the absence of gelatin trabeculae in the empty sponge and ASC–gelatin sponge groups. In all conditions, epithelialization was evident, with the formation of a clearly established Epidermal (E) layer (Figure 6). At this stage, CD31^+^ tubular vascular formations were observed within the granulation tissue, indicating the stabilization of newly formed BVs. Blood vessel formation was observed across all treatment groups; however, histological analysis revealed a significant increase in the number of CD31^+^ vascular structures in the allogeneic ASC–gelatin sponge-treated wounds compared to both the empty gelatin sponge and untreated controls (Figure 6). Software-assisted quantitative analysis of BV density confirmed a statistically significant difference between the allogeneic ASC–gelatin sponge group and empty gelatin sponge groups in the healing area (both superior to no treatment) (Figure 6). These findings demonstrate a pro-angiogenic effect of allogeneic ASCs embedded in a gelatin sponge, contributing to enhanced vascular content.

In conclusion, these observations established that the allogeneic ASCs in gelatin sponge increase the formation of granulation tissue and enhance vascularization, both essential for the healing of ischemic wounds. By day 7, the patch led to a significant increase in red blood cell infiltration within the granulation tissue, an early marker of improved tissue oxygenation. By day 21, histological analysis confirmed a statistically significant increase in blood vessel density in the ASC patch-treated wounds, reinforcing its pro-angiogenic effect. This is a critical parameter for ischemic conditions, where inadequate tissue oxygenation is a major barrier to healing. Given the rapid regenerative capacity of rodents compared to humans, the significant improvements observed in a short timeframe highlight the robust therapeutic potential of allogeneic ASC in a gelatin sponge scaffold. These findings strongly support its relevance for clinical applications in chronic ischemic wounds, such as diabetic foot ulcers and venous ulcers, where impaired vascularization is a leading cause of chronicity and poor healing outcomes.

### 2.7. Human ASCs in a Gelatin Sponge Upregulate MHC I Without Induction of Alloreactivity In Vitro

**Figure 7:**
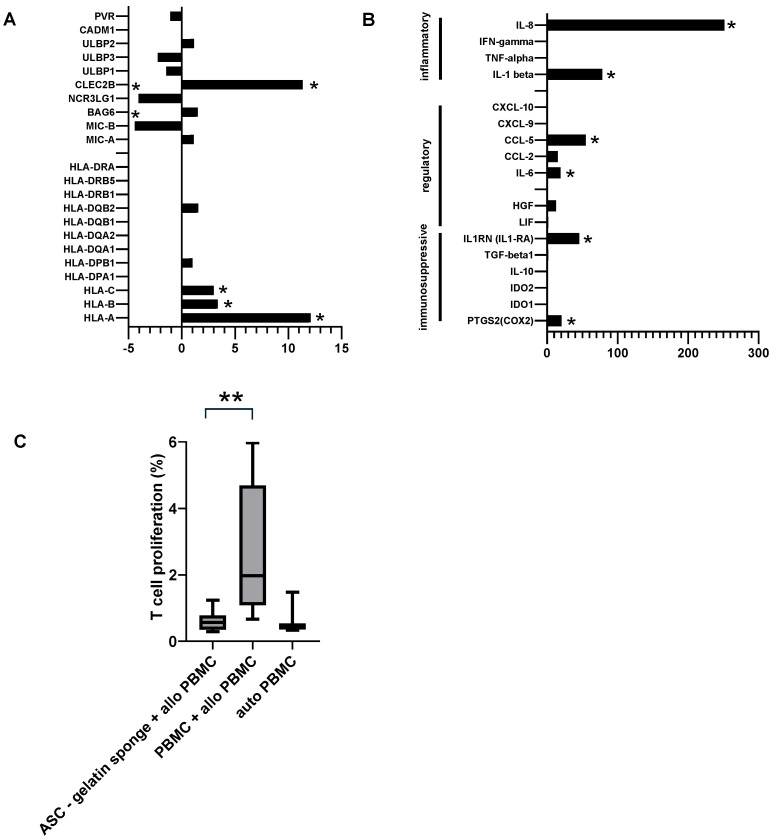
Human ASCs within a gelatin sponge modify their transcriptome and MHC molecules but do not induce alloreactivity in vitro. (**A**,**B**)—The transcriptome of human ASC, in gelatin generated from 3 independent cell lines, was assessed by microarray and compared to ASC from the same 3 donors grown in monolayer. Fold change analysis on MHC I and MHC II genes of the human polymorphic HLA system (**A**) or immune-related genes (**B**). Genes with an empty histogram were not detected. (**C**)—Mixed Lymphocyte Reaction, assessed by coculture of ASC or ASC extracted from a gelatin sponge with allogeneic PBMCs from 3 independent healthy donors. PBMCs from the same donor were used as the autologous stimulator cell control. * *p* < 0.05 (Benjamini.Hochberg) ** *p* < 0.01 (Mann–Whitney, GraphPad Prism 10).

To translate our findings in vitro from the rat model to the human system, we next investigated the immune behavior of human ASC incorporated into the same gelatin sponge scaffold. In contrast to rat ASCs, which were expanded in medium containing human platelet lysate (hPL) supplemented with fetal calf serum (FCS), human ASCs were cultured using hPL alone. These two formulations reflect species-specific optimization because rat ASCs exhibit poor proliferation in hPL-only conditions, and supplementation with a low concentration of FCS was required to achieve stable expansion (unpublished observations). In humans, by contrast, hPL alone provided sufficient growth factors to support ASC proliferation, as widely reported. For each species, the same culture conditions were consistently applied across all experimental steps. Transcriptomic profiling revealed a marked upregulation of HLA-A/B/C (MHC class I) or other receptors involved in alloreactivity (Figure 7A), consistent with the scaffold-induced activation previously observed in the rat setting. In parallel, the expression of several inflammatory mediators, including IL-1 and IL-8, as well as immunoregulatory factors such as IL1RN (IL-1 receptor antagonist) and CCL5, was increased (Figure 7B). Despite this gene expression profile, human ASC embedded in the gelatin sponge did not trigger any measurable allogeneic response in vitro, as demonstrated by the absence of responder T-cell proliferation in the mixed lymphocyte reaction (MLR) assay (Figure 7C). Together, these results mirror the in vivo observations in rats, where allogeneic ASC failed to elicit alloreactivity in vitro, and collectively support the feasibility and future testing of an allogeneic approach for ASC-based therapies in humans.

## 3. Discussion

The allogeneic ASC patch promoted the development of thicker, RBC-enriched granulation tissue and, by day 21, significantly increased vascular density relative to controls. These findings indicate that allogeneic ASCs in a gelatin sponge scaffold are both immunologically safe and therapeutically beneficial for enhancing vascularization and tissue repair in ischemic wounds. This is consistent with the established immunobiology of ASCs, which generally lack co-stimulatory molecules such as CD80, CD86, and CD40 required for full T-cell alloreactivity [39,40,41]. In addition, ASCs secrete immunoregulatory mediators—including indoleamine 2,3-dioxygenase (IDO) and prostaglandin E_2_ (PGE_2_)—that inhibit T-cell proliferation and activation [24,42,43]. Multiple studies have demonstrated that ASCs actively suppress alloreactivity in vitro [24,41], supporting our conclusion that even with a highly modified transcriptome profile in favor of angiogenicity and tissue repair, activated by the gelatin sponge, ASCs preserve their intrinsic lack of immunogenicity in an allogeneic situation. Thus, gelatin sponge-mediated activation does not compromise the immune tolerance properties of ASCs.

Gelatin embedding induced a robust paracrine activation of human ASCs, with increased secretion of several inflammatory cytokines and chemokines associated with early wound repair and angiogenesis. Importantly, this secretory profile did not imply enhanced immunogenicity. The factors upregulated in the ASC–gelatin constructs predominantly corresponded to innate inflammatory mediators that support vascular recruitment and matrix remodeling rather than T-cell activation. Consistent with this, ASCs did not express MHC class II molecules and did not stimulate allogeneic T-cell proliferation in the MLR, indicating that adaptive immune activation was not triggered despite the cytokine induction. Together, these findings suggest that the gelatin scaffold promoted a pro-regenerative ASC phenotype while preserving their low immunogenicity, a key requirement for allogeneic therapeutic applications. Although gelatin embedding significantly upregulated MHC class I expression on ASCs, this did not translate into increased immunogenicity in vivo or in MLR assays. This apparent discrepancy is consistent with the well-established immune-privileged features of ASCs, lacking expression of costimulatory molecules, rendering them inefficient at providing the secondary signals required for T-cell activation despite MHC class I upregulation. In addition, ASCs exert immunosuppressive effects through both contact-dependent mechanisms and the secretion of immunomodulatory mediators.

In vivo safety was confirmed by the absence of elevated levels of three acute phase proteins—alpha-1-acid glycoprotein, alpha-2-macroglobulin, and haptoglobin—all well-established biomarkers of immune reactivity in allogeneic transplantation rodent models [36,37]. The transient increase in these markers at day 3 across all experimental groups, including untreated animals, is most likely attributable to surgical trauma rather than immune activation against the implanted ASCs. Immunohistochemical analysis also revealed an absence of CD3^+^ T-cell infiltration in the implanted patch, a critical observation since early T-cell infiltration is a hallmark of allograft rejection. Together, systemic biomarker analysis and local histology confirm that the ASC–gelatin sponge construct does not elicit detectable alloreactivity in vivo.

The use of a relevant animal model was essential prior to introducing a controlled MHC mismatch. The clinical targets for this therapy are ischemic chronic wounds of diabetic or vascular origin—specifically diabetic foot ulcers (DFU) and venous leg ulcers (VLU)—which are characterized by severe tissue ischemia as a primary cause of delayed healing. Our chosen preclinical model, a rat footpad ischemic wound model, effectively induces tissue ischemia and delays wound closure, closely reproducing both the anatomical location and pathophysiological mechanisms of DFU/VLU in humans [31,44]. This model is widely recognized and validated: a systematic review and meta-analysis ranked it among the most clinically relevant for ischemic ulceration (score 5/9), second only to the same model combined with streptozotocin-induced hyperglycemia for DFU (score 9/9) [45]. Another comparative analysis of rodent, pig, and dog models reaffirmed that mice and rats are the most appropriate species for chronic wound studies, as their skin regeneration mechanisms best approximate those of humans [46]. While DFU represents a clinical cause of chronic wounds, it was not included in the present preclinical model. Our study was specifically designed to isolate ischemia as a primary driver of impaired healing, thereby modeling conditions such as VLU. Hyperglycemia is an important additional factor contributing to chronic wound pathology. However, separating ischemia from hyperglycemia allows a clearer assessment of the specific contribution of vascular insufficiency and of the therapeutic effect of ASC–gelatin sponge constructs in this context.

A central aim of this study was to recreate as far as possible in rodents the immunogenetic reality faced by “off-the-shelf” allogeneic ASC therapies in humans. In the clinic, a cell bank from a single donor would be used to treat many recipients with diverse HLA backgrounds, making MHC disparities unavoidable. To capture this, we transplanted ASC from two genetically distinct inbred strains expressing different MHC haplotypes, both with MHC I and MHC II (Lewis, RT1^l, and Brown Norway, RT1^n), into outbred Wistar rats (RT1^a). The use of a non-consanguine, outbred Wistar colony ensured minor antigen diversity among recipients, reflecting the heterogeneity of a human patient population. By imposing a defined MHC mismatch in a genetically varied recipient population, this model provides a stringent yet clinically relevant platform to evaluate both the efficacy and immunological safety of allogeneic ASC therapy. It reproduces the most consistent and unavoidable immunological barrier to such therapies in humans—class I—mediated alloimmunity—while allowing us to test whether the regenerative and angiogenic benefits of ASC can be achieved despite this challenge.

Histological analysis at day 7 showed that ischemic wounds treated with ASC–gelatin sponge patches developed thicker granulation tissue enriched in RBCs and devoid of T-cell infiltration. RBC enrichment likely reflects early tissue perfusion, a prerequisite for oxygen and nutrient delivery in ischemic wounds. The absence of immune cell infiltration, particularly T cells, indicates a reparative rather than immune rejection-mediated response. Over time, histological changes further supported the pro-healing activity of the ASC–gelatin sponge. Increased granulation tissue thickness and RBC content in early phases are indicative of improved oxygenation and healing potential, as RBC presence typically precedes mature vascular formation. By day 21, the ASC–gelatin sponge group displayed a significantly higher density of mature blood vessels compared to both empty sponge and untreated controls, suggesting that ASCs contribute to vascular assembly, maturation, and stabilization. This is consistent with our previous observations that ASCs activated by RGD within a gelatin scaffold stimulate endothelial cell migration, assembly, and stability and enhance vasculogenesis in human vascularized organoid models derived from pluripotent stem cells [15]. Enhanced vascular density at later stages is a key determinant of durable wound healing, especially in ischemic contexts where insufficient vascularization is a major barrier to treatment. Interestingly, while the gelatin sponge alone exhibited intrinsic pro-healing properties, promoting granulation tissue formation and contributing to early angiogenesis, the addition of ASCs provided distinct temporal advantages. ASC-loaded sponges enhanced early red blood cell infiltration on day 7, suggesting improved tissue perfusion prior to vessel formation, and significantly increased vascular density at day 21, supporting the maturation and stabilization of the neovascular network. This distinction is particularly relevant in chronic ischemic wounds, where durable and stable vascularization is essential to prevent relapses. Thus, while the scaffold itself initiates a regenerative response, ASCs appear to reinforce and sustain angiogenesis over time, supporting their added value in this context.

A deeper analysis of the cellular composition of the granulation tissue revealed that implantation of ASC–gelatin sponges did not alter the nature or proportion of cells infiltrating the granulation tissue. At all examined points, the constructs were populated by RBCs, neutrophils, macrophages, and fibrocytic cells, and image-analysis-based quantification showed that their relative distribution was comparable across untreated wounds, empty sponges, and ASC-loaded sponges (unpublished observations). These findings indicate that allogeneic ASC delivery does not elicit a heightened immune response and preserves the physiological inflammatory milieu of the healing wound.

The human MLR data further reinforced in vivo findings observed in the rat ischemic wound model. Consistent with the absence of T-cell infiltration and systemic alloreactivity in MHC-mismatched rats, gelatin-exposed human ASCs did not induce lymphocyte proliferation despite increased MHC class I expression. This cross-species concordance suggests that gelatin-mediated activation enhances pro-angiogenic function while preserving the intrinsic immune-modulatory properties of ASCs. Importantly, the human MLR assay directly addresses clinical allogeneic scenarios by evaluating functional immune responses rather than phenotypic markers alone, thereby complementing the rat transplantation data. Together, the lack of immunogenicity in both human in vitro and rat in vivo models supports the translational relevance of this approach and strengthens the rationale for developing gelatin-activated ASCs as standardized, off-the-shelf allogeneic cell therapies for ischemic wound repair. From a translational perspective, the use of allogeneic ASCs offers strong advantages over autologous approaches. Autologous ASC therapy is limited by donor-to-donor variability, differences in harvesting techniques and adipose tissue quality, very heavy procedures, high costs for cell line derivation, and impaired cell function in elderly or diabetic patients [2,21,22,23]. Allogeneic strategies using well-characterized, standardized ASC banks enable consistent product quality, scalable manufacturing, reduced production timelines, and lower costs. Moreover, off-the-shelf availability greatly facilitates clinical workflow and broadens patient access while retaining the low immunogenicity and immunosuppressive properties inherent to ASCs.

## 4. Materials and Methods

### 4.1. ASC Culture and Gelatin Sponge—ASC Patch Manufacturing

Rat ASCs were prepared from the subcutaneous fat of inbred Lewis or Brown Norway rats. ASCs were used between passage 2 and 5 and were cultured in Dulbecco’s Modified Eagle Medium DMEM (4.5 g/L glucose, L-Glutamine) (ThermoFisher, Waltham, MA, USA) supplemented with 10% of human platelet lysate (Stemulate, Cook Regentek, Bloomington, IN, USA), 10% of fetal calf serum (ThermoFisher, Waltham, MA, USA), and 1% penicillin and streptomycin (ThermoFisher, Waltham, MA, USA) at 37 °C and under 5% CO_2_. The details of rat ASC lines used are presented in Supplementary Table S1. Human ASCs were prepared from the subcutaneous fat of donors under informed consent of donors (ethical committee of the University Hospitals of Geneva, Switzerland (2020-01102, 15 September 2020)). The ASC lines used in this study were fully validated for their phenotype, multipotency, and regenerative potential. ASCs were cultured in Dulbecco’s Modified Eagle Medium (DMEM) with 4.5 g/L glucose and L-Glutamine, supplemented with 10% human platelet lysate (MultiPL100—Macopharma, Tourcoing, France) and 1% penicillin-streptomycin (ThermoFisher, Waltham, MA, USA) at 37 °C and under 5% CO_2_. To manufacture the rat or human ASC-gelatin sponge, a piece of sterile absorbable gelatin sponge USP Spongostan (standard, Ethicon, Raritan, NJ, USA) (1 cm (l) × 0.8 cm (w) × 0.7 cm (t)) was soaked in a suspension of ASCs at a final density of 6000 cells/mm3 in the ASC culture medium. The ASC-gelatin sponge was then cultured for 5 days in air/liquid interface conditions using a Millipore insert (polytetrafluoroethylene, 30 mm—0.4 µm pore) (Millipore, Burlington, MA, USA) floating on 1 ml of ASC culture medium in a 6-well plate.

### 4.2. Transcriptomics

Isolation of total RNA was performed by using the RNeasy kit from Qiagen (Hilden, Germany) according to the manufacturer’s instructions. RNA concentration was determined by a spectrometer (Thermo Scientific™ NanoDrop 2000, Waltham, MA, USA) and RNA quality was verified by the 2100 bioanalyzer (Agilent, Santa Clara, CA, USA). Rat or human microarray was performed with the ClariomTM S Assay (ThermoFisher, Waltham, MA, USA) using the Complete GeneChip^®^ Instrument System, Affymetrix. Transcript regulation was computed using TAC4.0.1.36 software (Applied Biosystems, Foster City, CA, USA) using the pheatmap package (https://cran.r-project.org/web/packages/pheatmap/index.html) with default settings. Enrichment of processes and pathways within the significantly upregulated or downregulated transcripts (fold change > and <2, FDR < 0.01) identified in the rat ASC-path compared to rat ASCs grown in monolayer was assessed using Metascape (www.metascape.org, accessed in 2024). The parameters used for the analysis were as follows: Organisms: Rattus Norvegicus; Input gene set: GO Biological Process; Min Overlap: 3; *p* value cutoff: 0.01; Min enrichment: 0.01.

### 4.3. Animal Experiments

The model of ischemic wound in the rat was the best available animal model of ischemic wounds and was performed as previously described [31,47]. Healthy Wistar outbred female rats of 250–300 g (Charles River Laboratories, Wilmington, MA, USA) were used and housed in an enriched environment. Before each surgery, all rats received buprenorphine to reduce pain. Rats were pre-anesthetized by inhalation of 5% isoflurane and anesthetized at a dose of 2%. Hairs were removed from the inguinal region using a mechanical shaver. All surgical procedures were performed under an operating microscope. Through a longitudinal incision made in the upper part of the left thigh, the external iliac and femoral arteries were dissected free along their entire length, from the common iliac to the saphenous artery, and a one cm-length artery was removed. Immediately after the arterial resection, a wound was created on the dorsal aspect of the feet in all animals by removing a full-thickness skin area of 1.2 × 0.8 cm. Treatments were applied a day after the surgery. To maintain the patches on the wound, a gutter of perforated silicone interface (Mepitel, Mölnlycke Health Care, Gothenburg, Sweden) was covered with a thin sheet of polyurethane (Opsite, Smith & Nephew, London, UK) and sutured around the wound. Rats’ weight was monitored for each rat during the entire period of study. Groups of 10 or 7 rats, depending on the experiments, were justified by ethical considerations and to ensure enough robust statistical analysis. When the rat altered the patch, animals were then excluded from statistical analysis. Randomization was used among the animals to define the experimental groups. Confounders (order of treatments) were minimized by the stability of the patch prior to application. Histological analysis was a blinded study, with anonymized samples for quantification of granulation thickness, RBC infiltration, and vessel density.

### 4.4. Mixed Lymphocyte Reaction

A mixed lymphocyte reaction (MLR) was performed using non-mitotically inactivated peripheral blood mononuclear cells (PBMCs) isolated from 3 healthy human donors by density-gradient centrifugation. Responder and stimulator PBMCs or human ASC from unrelated donors were co-cultured at a 10:1 ratio in complete RPMI-1640 medium supplemented with 10% fetal bovine serum. After 7 days of incubation, T-cell activation and proliferation were assessed by flow cytometry based on CellTrace™ Violet dilution, using the FlowJo software v10.90 (www.flowjo.com) and BD Accuri flow cytometer (BD Biosciences, San Jose, CA, USA). CD3^+^ T cells, labeled with an anti-human CD3-PE antibody, were gated in the FL-2 channel, and the % of proliferating cells was determined by measuring the number of gated cells having reduced CellTrace™ Violet fluorescence.

### 4.5. Quantification of Inflammatory Factors in Rat Serum

Alpha-1-acid glycoprotein, alpha-2-macroglobulin, and haptoglobin were measured in rat sera from each group at different time points between day 0 and day 21 post-treatment by using the Luminex Discovery Assay, Biotechne (R&D Systems, Minneapolis, MN, USA).

### 4.6. Statistical Analysis

Statistical analysis was performed using GraphPad Prism version 10.0 (Graphpad Software, La Jolla, CA, USA). *p*-values less than 0.05 were considered statistically significant and were indicated as follows: *: *p* < 0.05; **: *p* < 0.01; ***: *p* < 0.001 (non-parametric Mann–Whitney *t* test).

### 4.7. Immunofluorescence and Histological Colorations

For histological analyses, tissues were washed in PBS and fixed with a 4% paraformaldehyde solution for 20 min prior to dehydration and embedding in paraffin. Upon rehydration, slides (10 μm) were heated in citrate buffer 0.01 M and stained in PBS supplemented with bovine serum albumin 1% and Triton X-100 0.3% o/n at 4 °C with a rabbit IgG anti-CD3 (Abcam ab16669, 1/200, Cambridge, MA, USA) or goat anti-rat CD31 (R&D Systems, Minneapolis, MN, USA, AF3628, 1/200). For immunofluorescence (CD3), sections were stained with donkey anti-rabbit IgG-Alexa 555 antibody (Abcam ab 150074, 1/2000, Cambridge, MA, USA) and counterstained with DAPI and mounted in FluorSave medium (Calbiochem, San Diego, CA, USA). For immunohistochemistry (CD31), after washing in PBS, the sections were incubated for 10 min with a Biotinylated Pan-Specific Universal Antibody (PK-7800, Vector Laboratories, Newark, CA, USA), followed by another wash in PBS. The sections were then treated with a Streptavidin/peroxidase complex for 5 min and washed again in PBS. They were subsequently incubated in a peroxidase substrate solution (SK-4105, Vector Laboratories, Newark, CA, USA) until the desired staining intensity developed. After rinsing in tap water, the slides were stained for 5 min in a hematoxylin solution, followed by rinsing in running tap water and deionized water. The slides were then dehydrated in alcohol, cleared in xylene, and mounted. For hemalum/eosin coloration, paraffin-embedded tissue sections (10 µm) were mounted on glass slides, dried, and subsequently deparaffinized in Neoclear (Merck, Darmstadt, Germany) and rehydrated through a graded ethanol series (100%, 95%, 70%) to distilled water. Sections were then stained with Mayer’s hemalum (hematoxylin) for 5 min, rinsed in running tap water for 5–10 min, followed by an additional rinse in tap water. Slides were counterstained with eosin Y solution (1% in 95% ethanol with 0.5% acetic acid) for 1–2 min, dehydrated through graded ethanol, cleared in Neoclear, and coverslipped using a resin-based mounting medium. After staining procedures or colorations, the slides were imaged using an Axioscan microscope (Zeiss Axioscan.Z1, Zeiss, Oberkochen, Germany). The software Zen 3.11 and QPath 0.5.1 were used for the histological analysis and quantification workflow. For red blood cell area in granulation tissue and blood vessel quantification at days 14 and 21, a precise, constant, and anatomically defined Region of Interest (ROI) area was determined, ensuring consistency across all samples. Specifically, the ROI corresponded to the healing area delimited laterally by the three flexor digitorum tendons of the footpad and along the dorso-ventral axis between these tendons and the wound surface. QPath 0.5.1 was programmed to count the number of RBCs in the ROI. For blood vessel quantification, Zen 3.11 determined the number of vessels within the ROI.

## 5. Conclusions

This study demonstrates that allogeneic ASCs activated by a gelatin sponge do not acquire functional immunogenicity, maintain an immune-privileged profile both in vitro and in vivo, and promote early tissue perfusion as well as late-stage angiogenesis in ischemic wounds. The absence of local or systemic alloreactivity, together with robust pro-angiogenic effects, supports the clinical potential of the allogeneic ASC–gelatin sponge patch as a scalable and effective therapy for chronic ischemic wounds. Nevertheless, these findings were obtained in healthy young animal models, which cannot fully recapitulate the complex pathophysiology, metabolic alterations, and immune dysregulation characteristic of chronic wounds in patients. Further validation in more clinically relevant settings, including obesity, diabetic, or immunocompromised models, will be useful to strengthen translational relevance and support future clinical development.

## Data Availability

The original contributions presented in this study are included in the article/Supplementary Materials. Further inquiries can be directed to the corresponding author.

## Supplementary Materials

The following supporting information can be downloaded at https://www.mdpi.com/article/10.3390/ijms27083482/s1.
