# Supplementary material for: Gelatin Sponge-Embedded Adipose-Derived Stromal Cells Enable Allogeneic Application for Revascularization of Ischemic Wounds
Source: Int J Mol Sci. 2026 Apr 13;27(8):3482. doi: 10.3390/ijms27083482 (PMC13115642; doi:10.3390/ijms27083482)
Supplement: Supplementary file 1 [file ijms-27-03482-s001.zip › ijms-4201077-supplementary.pdf]

Supplementary Table S1. Rat ASC lines used

Strain	Brown Norway	Lewis	Wistar
RT1 Haplotype	RT1^n	RT1^l	RT1^a
Donor : gender	male	male	female
Manufacturing date	2024.06.20	2024.06.20	2024.01.31
Phenotype: CD31-, CD45-, CD29+ CD90+	99.40%	99.40%	99.20%
